# Personal

**Published:** 1917-08

**Authors:** 


### PERSONAL

Announcement of removal, travel, and other matters of interest are requested. Please report errors in the listing of any physician in the State and other directories, that we may co-operate with the State Society in securing a correct list.

Drs. A. W. Booth, LaRue Colegrove and Abraham Lande acted as committee of arrangements for an outing and dinner at the Cold Brook Club, July 19. Dr. Colegrove gave a talk on Bone Chips. Those in attendance consisted mainly of members of the Elmira Academy of Medicine and the Chemung County Society.

Dr. Guy S. Carpenter, Health Officer for Waverly, underwent an operation for gall stones at the People's Hospital of Sayre, July 15. To date, favorable reports have been received.

Dr. F. J. Moyer, Jr., of Lockport, is a candidate for nomination on the Republican ticket for member of the Assembly.

Major J. W. Grosvenor, M. D., of Buffalo, is taking a vacation of several weeks at Atlantic City.

Dr. René Gaultier, our associate editor, has been awarded the War Cross by the French Government.

**Medical Reserve Corps Appointments**: We have been unable to secure anything approaching a complete list for western N. Y. and request that appointees send in their names as soon as commissions are received and also report assignment to duty or camp service. The following appointments have come to our attention: H. C. Fairbanks of Tonawanda, Victor A. Tyrasinski, of N. Tonawanda, Ward B. Manchester, Batavia, Albert F. Ostwald, Buffalo, first lieutenants; Otto von Renner, Charles E. Long, A. L. Benedict, Buffalo, captains. Raymond C. Hill of Bath, Surgeon to the Soldiers' Home, has been ordered to Fort Benjamin Harrison, (Indianapolis).

The following officers of the Medical Reserve Corps are in training at Fort Benjamin Harrison: Benjamin Van Campen, Olean; Willard De Forrest Preston, Attica; S. M. Allerton and Don M. Hooks, Binghamton; John C. Grabau, Buffalo; Geo. S. Allen, Clyde; Walton Hovery, Fairport; James S. Allen, Geneva; Floyd W. Hayes, Jamestown; Harmon H. Ashley, Machias; R. M. Ballantyne, Manlius.

---

Dr. Edward Clark of Buffalo has been appointed Director of Child Hygiene in the N. Y. State Health Dept.

---

Dr. Julian P. Hill of West Ferry St., Buffalo, has moved his residence but not his office to Hamburg.

---

Dr. Ulysses B. Stein has returned to Buffalo, after a residence of a few years in Newfane, and has opened an office at the corner of Delaware Ave. and Chippewa St.

---

Dr. Maud J. Frye of Buffalo, announces the removal of her office to 211 Highland Ave., practice limited to Diseases of Women.

---

Dr. J. D. Tillotson of Rochester has moved from 796 Clinton Ave. to 16 Champlain St.

---

Dr. G. C. Fisk has moved from Mt. Morris to 108 Pine St., Buffalo.

